# PIntron: a fast method for detecting the gene structure due to alternative splicing via maximal pairings of a pattern and a text

**DOI:** 10.1186/1471-2105-13-S5-S2

**Published:** 2012-04-12

**Authors:** Yuri Pirola, Raffaella Rizzi, Ernesto Picardi, Graziano Pesole, Gianluca Della Vedova, Paola Bonizzoni

**Affiliations:** 1 Dipartimento di Informatica Sistemistica e Comunicazione, Univ. degli Studi di Milano-Bicocca, Milano, 20126, Italy; 2 Centro Ricerche e Studi Agroalimentari, Parco Tecnologico Padano, Lodi, 26900, Italy; 3 Dipartimento di Biochimica e Biologia Molecolare "E. Quagliariello", Univ. degli Studi di Bari, Bari, 70126, Italy; 4 Istituto di Biomembrane e Bioenergetica, Consiglio Nazionale delle Ricerche, Bari, 70126, Italy; 5 Dipartimento di Statistica, Univ. degli Studi di Milano-Bicocca, Milano, 20126, Italy

## Abstract

**Background:**

A challenging issue in designing computational methods for predicting the gene structure into exons and introns from a cluster of transcript (EST, mRNA) sequences, is guaranteeing accuracy as well as efficiency in time and space, when large clusters of more than 20,000 ESTs and genes longer than 1 Mb are processed. Traditionally, the problem has been faced by combining different tools, not specifically designed for this task.

**Results:**

We propose a fast method based on *ad hoc *procedures for solving the problem. Our method combines two ideas: a novel algorithm of proved small time complexity for computing spliced alignments of a transcript against a genome, and an efficient algorithm that exploits the inherent redundancy of information in a cluster of transcripts to select, among all possible factorizations of EST sequences, those allowing to infer splice site junctions that are largely confirmed by the input data. The EST alignment procedure is based on the construction of *maximal embeddings*, that are sequences obtained from paths of a graph structure, called embedding graph, whose vertices are the *maximal pairings *of a genomic sequence *T *and an EST *P*. The procedure runs in time linear in the length of *P *and *T *and in the size of the output.

The method was implemented into the PIntron package. PIntron requires as input a genomic sequence or region and a set of EST and/or mRNA sequences. Besides the prediction of the full-length transcript isoforms potentially expressed by the gene, the PIntron package includes a module for the CDS annotation of the predicted transcripts.

**Conclusions:**

PIntron, the software tool implementing our methodology, is available at http://www.algolab.eu/PIntron under GNU AGPL. PIntron has been shown to outperform state-of-the-art methods, and to quickly process some critical genes. At the same time, PIntron exhibits high accuracy (sensitivity and specificity) when benchmarked with ENCODE annotations.

## Background

A key step in the post-transcriptional modification process is called *splicing *and consists of the excision of the intronic regions of the premature mRNA (pre-mRNA) while the exonic regions are then reconnected to form a single continuous molecule, the mature mRNA. A complex regulatory system mediates the splicing process which, under different conditions, may produce *alternative *mature mRNAs (also called transcript isoforms) starting from a single pre-mRNA molecule. Alternative Splicing (AS), i.e. the production of alternative transcripts from the same gene, is the main mechanism responsible for the expansion of the transcriptome (the set of transcripts generated by the genome of one organism) in eukaryotes and it is also involved in the onset of several diseases [1].

A great extent of work has been performed to solve two basic problems on AS: characterizing the exon-intron structure of a gene and finding the set of different transcript isoforms that are produced from the same gene. Some computational approaches, based on transcript data, for these crucial problems have been proposed; indeed good implementations are available [2-9]. Recently, some tools related to the problem, but limited to the specific task of predicting splice junctions from Next-Generation Sequencing (NGS) data, have been designed [10-13]. These tools are computationally intensive and would require a post-processing step to filter the correct data that can be related to the alternative exon-intron structure of a gene. Moreover, the literature provides efficient solutions for computing a specific spliced alignment of an EST against the genome (for example Exonerate [14], GMAP [15] and Spaln [16]). However these tools are designed to compute only spliced alignments and not to directly provide the complete exon-intron structure of a gene and its full-length isoforms.

In this paper we provide a specifically designed algorithm - efficient from both a theoretical and an empirical point of view - to predict the exon-intron structure of a gene from general transcript data that is optimal with respect to constraints derived by the input data. The algorithm is implemented in a tool, called PIntron. Similarly as recent programs [5,7], PIntron is a method for exon-intron structure prediction, but differently from these tools is able to efficiently process complex genes or genes associated with a large cluster of ESTs. Indeed, the accurate prediction of the exon-intron structure of a gene is a computational hard task when the redundancy of the information given by EST data must be taken into account. More precisely, combinatorial methods for the problem are highly accurate when they are able to combine two different steps: (1) producing putative spliced alignments of ESTs against the gene region and (2) selecting among the different putative spliced alignments of each EST those confirming the same gene structure under some optimization criteria. This second step has been proved to be NP-hard [17] thus requires efficient heuristics.

On the other hand, finding putative spliced alignments (first phase) could be a challenging task when more than one alignment exists for the same transcript. Indeed, for instance, there could be different possible splicing junctions between consecutive exons because of the presence sequencing errors or repeated genomic regions. As a consequence, choosing the correct spliced alignment of a single EST sequence requires to perform a multiple comparison between several spliced alignments of all the EST sequences in order to find the ones that support a common putative gene structure. In [18] a detailed discussion of this issue is provided.

## Methods

In this paper we show how to efficiently solve the integration of the two steps of finding the (possibly different) spliced alignments of a cluster of transcripts and using them to compute a common gene structure.

Overall, our new combinatorial method for exon-intron structure prediction can be summarized as a four-stage pipeline where we:

1. Compute and implicitly represent all the spliced alignments of a transcript sequence (EST or mRNA) against a genomic reference sequence by a novel graph representation, called *embedding graph*, of the common substrings of the transcripts and the genome. In this paper we provide efficient algorithms for building and, subsequently, visiting the embedding graph.

2. Filter all biologically meaningful spliced alignments. This step is performed with a carefully tailored visit of the embedding graph.

3. Reconcile the spliced alignments of a set of correlated transcript sequences into a maximum parsimony consensus gene structure. To complete this task we use the *Minimum Factorization Agreement (MFA) *approach [17] applied to the data produced by the previous step. Indeed, the MFA approach gives an effective method to amalgamate some spliced alignments into a consensus gene structure (notice that an EST sequence only provides information on a partial region of the whole gene).

4. Extract, classify, and refine the resulting introns in order to provide a putative gene structure supported by transcript evidences.

We point out that our implementation also has a fifth step where it predicts a set of full-length isoforms by employing the graph-based method in [19].

**Figure 1:**
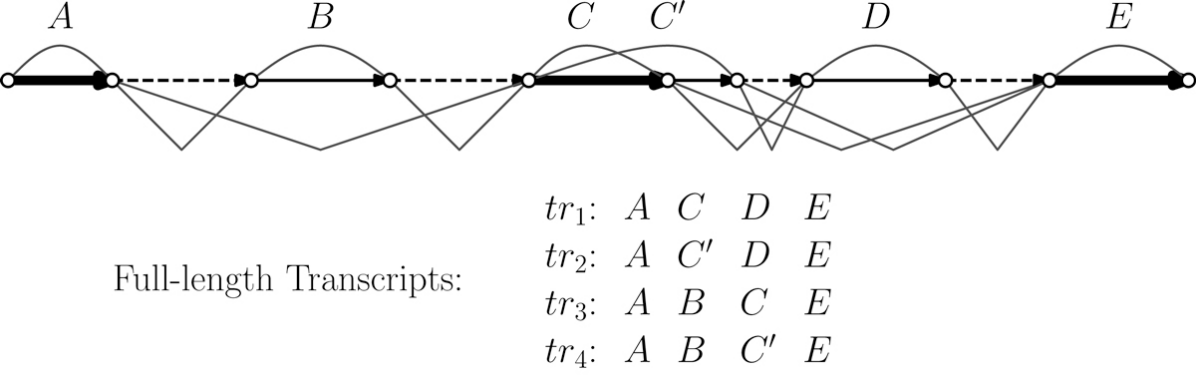
**The colored directed graph representing a gene structure**. The represented gene structure, induced by compositions, is composed by 6 genomic exons: *A*, *B*, *C*, *C'*, *D*, *E*. Dashed edges represent noncoding regions, bold edges represent regions included into all the gene isoforms, and the remaining normal edges represent regions that are both coding and noncoding (i.e. are included into some gene isoform and are retained as a part of an intron into some other isoform). For clarity, we indicated an exon with a curve above the graph, and an intron with two connected segments below the graph. Observe that *C *and *C' *are competing exons, while exons *B *and *D *are cassette exons.

Our method computes a consensus gene structure minimizing the number of exons, called maximum parsimony consensus gene structure. Such a structure is strictly associated to a set of spliced alignments for each sequence in the cluster of transcript data that is also output by our algorithm. Informally, a gene structure (depicted in Figure 1) is the description of the location of coding (exon) and noncoding (intron) regions along the genomic sequence. Due to alternative splicing events, such as exon skipping, intron retention and competing exons, a portion of the genomic sequence could be both coding and noncoding with respect to different transcripts.

In this paper, we will evaluate all steps of the pipeline. Accuracy and efficiency of PIntron have been assessed by an experimental comparison with ASPic [20] and Exogean [21]. The experimental results show that PIntron is much faster than ASPic and competitive with Exogean. PIntron scales much better than Exogean (in terms of execution time) when processing genes with a large number of transcript sequences. The predictions made by PIntron are more accurate than those by ASPic and Exogean. Moreover, PIntron is the only tool that is able to successfully complete all genes that have been considered. Finally, our results indicate that PIntron also improves the reconstruction of exact transcripts when compared with the other two tools.

In this experimental comparison, we focused on human genes given their excellent annotation status. However, PIntron has been conceived to facilitate genome annotation in a variety of organisms in which expressed sequences as well as the reference genome are available. Given the experimental results we summarized above, our program enables the investigation of the impact of alternative splicing on large-scale.

The rest of this section is devoted to present each algorithmic step of our four-stage pipeline.

### Implicit computation of spliced alignments

The first stage of our gene structure prediction method computes the set of all possible spliced alignments of a transcript (EST or mRNA) sequence against the genomic sequence.

A spliced alignment is a particular kind of alignment that takes into account the effects of the excision of the intronic regions during the RNA splicing process. The spliced sequence alignment problem requires to compute, given a sequence *P *(the EST or the mRNA) and a reference sequence *T *(the genomic sequence), two sets *F_P _*= {*f*_1_,..., *f_k_*} and $F_{T}=\left\{f_{1}^{\prime },...,f_{k}^{\prime }\right\}$ of strings such that *P *= *f*_1 _... *f_k_*, $T=pf_{1}^{\prime }i_{1}f_{2}^{\prime }i_{2}\cdots f_{k-1}^{\prime }i_{k-1}f_{k}^{\prime }s$, and for each *i*, the edit distance between $f_{i}$ and $f_{i}^{\prime }$is small. The sequence of pairs $\left(f_{i},f_{i}^{\prime }\right)$ is called *composition *of *P *on *T*, each factor *f_i _*is called spliced *sequence factor *(or EST factor), and each $f_{i}^{\prime }$ is called *genomic factor *(or exon). Allowing a small edit distance between the two factors is justified by the fact that EST data contain mismatches (deletions and insertions) against the genome because of sequencing errors and polymorphisms. Unfortunately, this also makes computationally harder the spliced alignment problem, especially when the transcript and the genomic sequence are large.

In our novel alignment method, we exploit the small edit distance between each pair $\left(f_{i},f_{i}^{\prime }\right)$ of corresponding factors: in fact, in this case, there must exist a sequence of some sufficiently long common substrings of the EST factor *f_i _*and the genomic factor $f_{i}^{\prime }$. We call the sequence of the occurrences of perfectly matching substrings an *embedding *of the EST sequence *P *in the genomic sequence and, clearly, it reveals the basic "building blocks" of the spliced alignment. Our alignment algorithm is based on the construction of a compact and implicit representation of all the embeddings by means of a graph called *embedding graph*. Such a graph can be efficiently computed from the EST sequence *P *and the genomic sequence *T *in time *O*(|*P*|+ |*T*|+ |*V*|^2^), where *V *is its vertex set, and it can be used in the second stage of our pipeline in order to efficiently enumerate all the biologically meaningful compositions.

In the following we detail the notion and construction of the embedding graph. Let us first recall, that according to the traditional notation, given a string *S *= *s*_1_*s*_2 _... *s_q_*, we denote with |*S*| its length and with *S*[*i*, *j*] the substring *s_i_s_i_*_+1 _... *s_j_*.

A fundamental notion is that of *pairing *of two strings. More formally, a *pairing *(*p*, *t*, *l*) of two sequences *P *and *T *(which generalizes the notion of pair of a sequence [22]) represents the positions *p *on *P *and *t *on *T *of a common substring *P*[*p*, *p*+*l *- 1] = *T*[*t*, *t*+*l *- 1] of *P *and *T*. In other words, a pairing (*p*, *t*, *l*) represents a common substring *x *of *P *and *T*, called *factor *induced by the pairing, such that *x *is of length *l *starting in positions *p *and *t *on *P *and *T *respectively. The positions *p *and *t *are called starting positions, while *p *+ *l *and *t *+ *l *are called ending positions.

We say that a pairing $v_{1}=(p_{1},t_{1},l_{1})$ is *contained *in a pairing $v_{2}$ (in short $v_{\text{1}}≼v_{\text{2}}$) if the positions $p_{1}$ and $t_{1}$ of $v_{1}$ can be extended to the left or to the right on both the sequences *P *and *T *in order to obtain $v_{2}$. Clearly, the factor induced by *v*_1 _is a substring of the factor induced by $v_{2}$. Moreover, we say that *v*_1 _is a *prefix-pairing *(*suffix-pairing*, resp.) of $v_{2}$ iff $v_{\text{1}}≼v_{\text{2}}$ and $v_{1}$ shares the same starting (ending, resp.) positions on  P and  T of $v_{2}$. This fact implies that the factor induced by $v_{1}$ is a prefix (suffix, resp.) of the factor induced by $v_{2}$on *P *and *T*. A pairing *v *is *maximal *if and only if there does not exist a distinct pairing containing  v. In other words, *v *is maximal if and only if the common factor induced by  v cannot be "extended" neither to the left nor to the right on both *P *and *T*.

**Figure 2:**
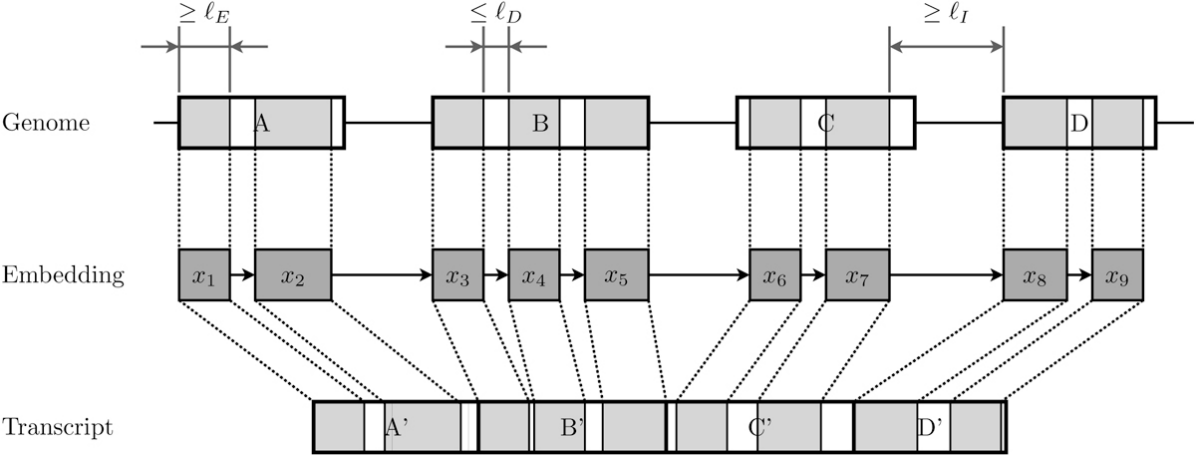
**An embedding and its relationships with the genome and a transcript**. The *x*_1_,...,*x*_9 _are substrings shared by the genome and the transcript corresponding to pairings. Each common substring (pairing) is longer than a fixed threshold ℓ*_E_*. Intuitively, when the distance (measured on the genome) between two consecutive pairings is smaller than ℓ*_D _*then we assume that those pairings belong to the same exon. When the same distance is larger than ℓ*_I _*then those pairings belong to different exons.

A sequence of non-overlapping pairings (i.e. pairings that represent non-overlapping occurrences of common substrings) is called an *embedding *(see Figure 2). Given two embeddings $\varepsilon =\langle v_{1},\ldots ,v_{n}\rangle$ and $\varepsilon^{\prime }=\langle v_{1}^{\prime },\cdots \;,v_{m}^{\prime }\rangle$, then  ε is contained in ε' (in short $\varepsilon ≼\varepsilon^{\prime }$) if and only if for each $v_{i}$ in  ε there exists a pairing $v_{j}^{\prime }$ in ε' such that $v_{i}≼v_{j}^{\prime }$. Given the set  of the embeddings of *P *in *T*, we say that ε∈E is *maximal *iff there does not exist $\varepsilon^{\prime }\in E$, ε≠ε', such that $\varepsilon ≼\varepsilon^{\prime }$.

Not all embeddings induce a biologically meaningful composition. For example, an embedding made of several short pairings "scattered" along the genome cannot be considered a valid spliced alignment. In order to restrict embeddings to be useful for building a spliced alignment, we fix three parameters ℓ*_E_*, ℓ*_D _*and ℓ*_I_*. Intuitively, the parameter ℓ*_E _*is the minimum length of a pairing, ℓ*_D _*limits the maximum number of consecutive mismatches that can appear in a single exon, and ℓ*_I _*represents the minimum length of an intron. Then a *representative embedding *is a maximal embedding $\varepsilon =\langle v_{1},\ldots ,v_{m}\rangle$ such that *l_i _*≥ ℓ*_E_*, *p_i_*_+1 _-*p_i _*- *l_i _*≤ ℓ*_D_*, and either (*i*) |*t_i_*_+1 _-*t_i _*- (*p_i_*_+1 _- *p_i_*)| ≤ ℓ*_D _*or (*ii*) *t_i_*_+1 _- *t_i _*- (*p_i_*_+1 _- *p_i_*) ≥ ℓ*_I _*is true. It is easy to see that only representative embeddings might induce a biologically plausible composition.

Indeed, a careful choice of the three parameters ℓ*_E_*, ℓ*_D _*and ℓ*_I _*allows to recover a spliced alignment of *P *in *T *with a fixed (small) error rate from some representative embeddings. Therefore, we propose the problem of finding all representative embeddings of *P *in *T*, formalized as the REPRESENTATIVE EMBEDDING problem (RE), where we are given a pattern *P*, a text *T*, and three parameters ℓ*_E_*, ℓ*_D _*and ℓ*_I_*. The goal is to compute the set $\varepsilon_{r}$ of the representative embeddings of *P *in *T*.

In this first stage of the pipeline, we tackle the RE problem by using the embedding graph defined as follows.

**Definition **(Embedding Graph). Given a pattern *P *and a text *T*, the *embedding graph *of *P *in *T *is a directed graph *G *= (*V*, *E*) such that the vertex set *V *is the set of maximal pairings of *P *and *T *that are longer than ℓ*_E_*. Two pairings *v*_1 _= (*p*_1_, *t*_1_, *l*_1_) and *v*_2 _= (*p*_2_, *t*_2_, *l*_2_) are connected by an edge (*v*_1_, *v*_2_) ∈ *E *if and only if: (i) *p*_2 _- (*p*_1 _+ *l*_1_) ≤ ℓ*_D_*, and (ii) |*t*_2 _- *t*_1 _- (*p*_2 _- *p*_1_)| ≤ ℓ*_D _*or *t*_2 _- *t*_1 _- (*p*_2 _- *p*_1_) ≥ ℓ*_I_*.

Basically the conditions of the definition of Embedding Graph ensure the following crucial property: Two maximal pairings $v_{1}$ and $v_{2}$ are connected by an edge in the embedding graph if and only if there exists a representative embedding  ε in which there are two consecutive pairings $v_{i}^{\prime }$ and $v_{i+1}^{\prime }$ such that $v_{i}^{\prime }$ is contained in $v_{1}$ and $v_{i+1}^{\prime }$ is contained in $v_{2}$.

We will use this property to build representative embeddings from an embedding graph. Observe that such a property derives from the maximality of the representative embeddings and from the uniqueness of the maximal pairing containing a pairing which belongs to a representative embedding.

We designed an algorithm that builds the embedding graph of a pattern *P *and a text *T *in time *O*(|*T*|+ |*P*| + |*V*|^2^). The algorithm is composed of two steps. In the first step, the vertex set *V *is computed by visiting the suffix tree of the text *T*. This step requires *O*(|*T*|) time for the suffix tree construction and *O*(|*P*| + |*V*|) time for the computation of maximal pairings. In the second step, edges are then computed by checking the conditions of the definition of embedding graph on each pair of maximal pairings, leading to an *O*(|*V*|^2^) procedure. Since the number of maximal pairings is usually very small compared to the length of *P *and *T*, the embedding graph construction procedure is efficient even on large patterns *P *and texts *T*.

### Extraction of relevant spliced alignments

The next stage of our pipeline is devoted to analyzing and mining the embedding graph to compute the representative embeddings that also induce *distinct *biologically meaningful compositions. Indeed, it must be pointed out that different representative embeddings can induce the same compositions or spliced alignments. Algorithm **ComputeCompositions **is a two-step procedure. Initially it extracts a subset of representative embeddings by performing a visit of the embedding graph. Then the algorithm computes the compositions by merging consecutive pairings that are separated by short gaps.

#### Embedding graph visit

The first step of **ComputeCompositions **is a recursive visit of the embedding graph starting from a subset of vertices that we call *extended sources*.

Such a procedure visits the embedding graph examining and extracting only pairwise-distinct representative embeddings that are biologically meaningful (for example with respect to the length of gaps representing errors or introns). More precisely, the visit of a vertex $v_{k}$ from the extended source *s *reconstructs the set  of biologically meaningful representative embeddings that are induced by the path $P=\langle s,v_{1},...,v_{k}\rangle$ traversed during the visit of the embedding graph.

**Figure 3:**
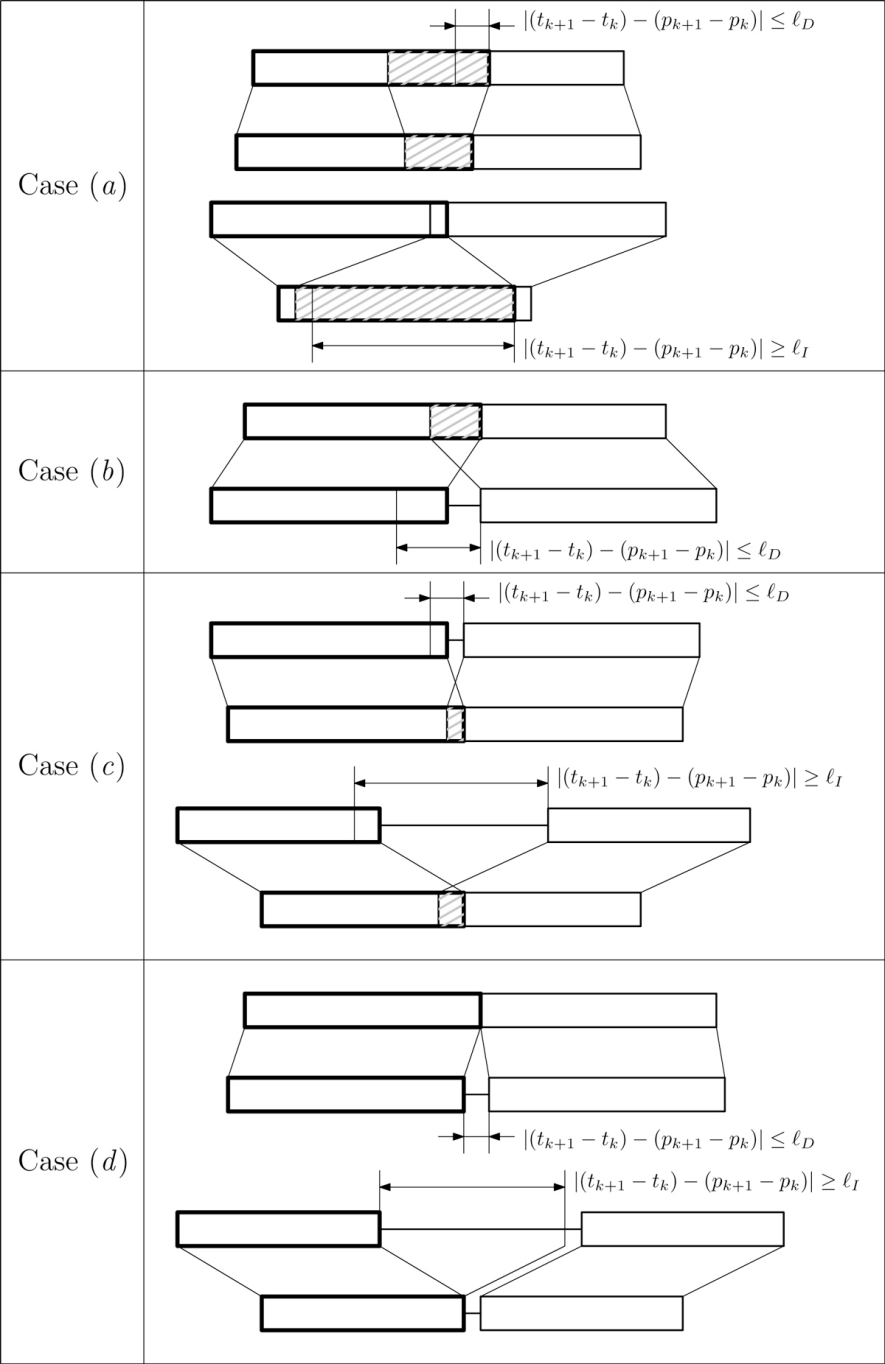
**Possible relative positions of two maximal pairings connected by an embedding graph edge**. The figure presents the possible configurations of relative positions of two maximal pairings *e_k _*= (*p_k_*, *t_k_*, *l_k_*) and *v_k_*_+1 _= (*p_k_*_+1_, *t_k_*_+1_, *l_k_*_+1_) connected by an embedding graph edge (*e_k_*, *v_k_*_+1_). Each box represents a common maximal factor on *T *(top) and *P *(bottom) of a maximal pairing. Each maximal pairing is represented by two boxes connected by lines (boxes representing *e_k _*are in bold). For each case, *t_k _*corresponds to the left border of the upper bold box, *p_k _*is the left border of the lower bold box, *t_k_*_+1 _is the left border of the upper normal box, and *p_k_*_+1 _is the left border of the lower normal box. Distance |(*t_k_*_+1 _- *t_k_*) - (*p_k_*_+1 _- *p_k_*)| has been represented by a double ended arrow, while factor overlaps are highlighted by grey shades. Four possible cases are presented: *(a) e_k_*, *v_k_*_+1 _overlap on both *T *and *P*, *(b) e_k_*, *v_k_*_+1 _overlap on *T *but not on *P *, *(c) e_k_*, *v_k_*_+1 _overlap on *P *but not on *T*, and *(d) e_k_*, *v_k_*_+1 _do not overlap neither on *T *nor on *P*.

We will now explain the main steps of the procedure. During the visit of vertex *v_k_*, we examine each outgoing edge (*v_k_*, *v_k_*_+1_) and we "extend" each embedding $\varepsilon =\langle e_{1},\ldots ,e_{k}\rangle$ of . How the extension is performed depends on the relative position, on *P *and *T*, of *e_k _*in  ε and the new vertex *v_k_*_+1 _that are depicted in Figure 3. In the exposition of the different possible cases, let *e_k _*= (*p_k_*, *t_k_*, *l_k_*) and *v_k_*_+1 _= (*p_k_*_+1_, *t_k_*_+1_, *l_k_*_+1_). Observe that given two pairings that are connected by an edge in the embedding graph, the corresponding factors might be overlapping in the text or in the pattern. To simplify the notation, in the following we identify a pairing with the factor it induces.

**Case (a)**. Factors *e_k _*and *v_k_*_+1 _overlap on both *T *and *P*. Two different sub-cases must be analyzed. The first case occurs when the distance between the two initial positions of the factors *e_k _*and *v_k_*_+1 _on *P *differs from the same distance on *T *of a value (positive or negative) less than ℓ*_D_*, while the second case occurs when such a distance differs of a value greater than ℓ*_I_*. If the first case occurs when |(*t_k_*_+1 _-*t_k_*) - (*p_k_*_+1 _- *p_k_*)| ≤ ℓ*_D _*then the two pairings may belong to the same factor of the induced composition. Thus, the algorithm replaces pairing *e_k _*in  ε with the shortest maximal prefix-pairing $e_{k}^{\prime }$ of *e_k _*and the longest maximal suffix-pairing *e_k_*_+1 _of *v_k_*_+1 _such that they do not overlap and that both $e_{k}^{\prime }$ and *e_k_*_+1 _are at least ℓ*_E _*long. The second case occurs when (*t_k_*_+1 _- *t_k_*) - (*p_k_*_+1 _- *p_k_*) ≥ ℓ*_I_*. This case deserves a special discussion from the biological point of view since it could be related to an intron as well as to a tandem repeat in *T*. Then factor *e_k _*could be extended to include the repetition in *v_k_*_+1 _to produce a unique factor (exon) of the embedding  ε.

**Case (b)**. Factors induced by *e_k _*and *v_k_*_+1 _overlap in *T *but not in *P*. This case is equivalent to the first sub-case of Case (a).

**Case (c)**. Factors *e_k _*and *v_k_*_+1 _overlap in *P *but not in *T*. Just as in Case (a) two different sub-cases must be analyzed, that is either |(*t_k_*_+1 _- *t_k_*) - (*p_k_*_+1 _-*p_k_*)| ≤ ℓ*_D _*or *t_k_*_+1 _-*t_k _*- (*p_k_*_+1 _- *p_k_*) ≥ ℓ*_I_*. The first case is solved as in Case (a). Notice that when the second subcase occurs then the splice site placement is ambiguous because a suffix of the donor exon is equal to a prefix of the acceptor exon. Also in this case, basic biological criteria are used to reduce the impact of the ambiguity.

**Case (d)**. Factors *e_k _*and *v_k_*_+1 _do not overlap neither in *P *nor in *T*. Let *G_T _*and *G_P _*be the two substrings which separate *e_k _*and *v_k_*_+1 _in *T *and *P*, respectively. Since *G_P _*and *G_T _*do not form a pairing, they must contain a certain number of mismatches; we must determine if they support the possibilities that (i) *e_k _*and *v_k_*_+1 _are part of the same factor or (ii) there is an intron between *e_k _*and *v_k_*_+1_. Similarly to Case (a), two different sub-cases may arise. If |(*t_k_*_+1 _- *t_k_*) - (*p_k_*_+1 _- *p_k_*)| ≤ ℓ*_D_*, then *e_k _*and *v_k_*_+1 _might belong to the same factor of the induced composition. More precisely, *e_k _*and *v_k_*_+1 _belong to the same factor if the edit distance between *G_T _*and *G_P _*is below a certain threshold - in which case *v_k_*_+1 _is added to embedding  ε, otherwise the edge is discarded from the visit. Instead, if *t_k_*_+1 _-*t_k _*- *p_k_*_+1 _+ *p_k _*≥ ℓ*_I_*, the two pairings are separated by an intron, and we must determine the splice sites of such an intron. In this case, the algorithm computes a prefix $G_{T}^{\prime }$ and a suffix $G_{T}^{\prime \prime }$ of *G_T _*that minimize the edit distance between *G_P _*and the concatenation of $G_{T}^{\prime }$ and $G_{T}^{\prime \prime }$. Also in this case, if the resulting edit distance is larger than an acceptable threshold, the edge (*v_k_*, *v_k_*_+1_) is discarded, otherwise *v_k_*_+1 _is added to  ε. Notice that computing the edit distance is not too expensive, since all strings involved are no longer than 2ℓ*_D_*.

The definition of embedding graph allows the presence of directed cycles, which potentially might be troublesome. However, we claim that the embeddings, computed from a path  containing a cycle , would induce compositions with essentially the same set of factors of the compositions induced by the embeddings computed from the visit of the simple path P\C. The visit performed in the first step of algorithm. **ComputeCompositions** guarantees that each possible representative embedding is analyzed. However, the biological criteria that we employ allow to consider only pairings belonging to biologically meaningful embeddings. Since the visit computes pairwise-distinct representative embeddings and every case presented above requires *O*(1) time, the overall computational complexity of the visit is clearly bounded by $O\left(\sum_{\varepsilon \in E}|\varepsilon |\right)$, that is the total size of the representative embeddings that have been computed during the visit.

#### Composition reconstruction

The set  of representative embeddings computed by the visit of the embedding graph directly leads to a set *C *of compositions. In fact, the visit guarantees that two consecutive pairings of a representative embedding are either separated by a small gap due to errors or by a large gap representing an intron of the spliced alignments. Hence, the algorithm simply merges into a factor a sequence of factors induced by consecutive pairings *v_k _*= (*p_k_*, *t_k_*, *l_k_*) and *v_k_*_+1_= (*p_k_*_+1_, *t_k_*_+1_, *l_k_*_+1_) separated by small gaps, that is |*t_k_*_+1 _- *t_k _*- *p_k_*_+1 _+ *p_k_*| ≤ ℓ*_D_*. Finally, the composition is retained if the edit distance between each EST factor and the corresponding genomic factor is not larger than a fixed acceptable threshold.

### Building a gene structure

The first two stages of our pipeline are applied separately to each transcript sequence *P_i _*of the input data (a genomic sequence *T *and a set  of transcripts) computing a set *C*(*P_i_*) of biologically meaningful compositions for each *P_i_*. The main goal of the third stage is to extract a composition for each transcript that explains the putative gene structure. As stated before, informally a gene structure is the description of the location of coding and noncoding regions along the genomic sequence, where by a coding region we mean an exon and by noncoding region we mean an intron. Note that the boundaries between an exon and an intron is called splice junction or splice site.

We aim to produce a maximum parsimony *consensus gene structure *for  which consists of a minimum set of genomic exons or coding regions compatible with a high quality composition *C_i _*for each transcript data *P_i_*. The minimization criteria is used to avoid overprediction of splice junctions. For this task we propose a formalization of the problem of finding a putative gene structure, called CONSENSUS GENE STRUCTURE problem (CG) and discuss a solution of this problem. The input of the CG problem consists of a set *C*(*P_i_*) of compositions for each transcript *P_i _*in a set  and a finite ordered set *F *= 〈*f*_1_, *f*_2_,..., *f*_|*F*|_〉 of genomic factors induced by the compositions in ∪*C*(*P_i_*). Ordering of factors is assigned by considering their left splice junctions. Then CG asks for the minimum cardinality subset *F' *of *F *such each *P_i _*has a composition with all genomic factors in *F'*. In other words *F' *is the minimum set of exons explaining a spliced alignment of each EST data.

Now, the CG problem can be faced by using the approach [17] called *Minimum Factorization Agreement *(MFA). More precisely, we use the MFA problem to compute a gene structure minimizing the number of exons.

Let us recall the definition of the MFA problem. Let *F *= 〈*f*_1_, *f*_2_,..., *f*_|*F*|_〉 be a finite ordered set of sequences over alphabet Σ, called *factors *and let *S *be a set of sequences over alphabet Σ. Given a sequence *s *∈ *S*, a *factor-composition *(*f-composition *in short) of *s *consists of the sequence $f=\langle f_{i_{1}},f_{i_{2}},\cdots \;,f_{i_{n}}\rangle$ such that $s=f_{i_{1}},f_{i_{2}},\cdots \;,f_{i_{n}}$ and *i_j _< i_j_*_+1 _for 1 ≤ *j < n*. Then the set $\left\{f_{i_{1}},f_{i_{2}},\cdots \;,f_{i_{n}}\right\}$ is called the *factor set *of *f *and is denoted as *F*(*f*). While the notion of f-composition depends on the set of factors, such set of factors is usually clear from the context and is therefore omitted. Please notice that a sequence *s *can admit different f-compositions: thus let *F*(*s*) be the set of compositions of *s*. Moreover, by extension, we will denote by *F*(*S*) the set ∪*_s_*_∈_*_S_F*(*s*) of all f-compositions of a set *S *of sequences. Given a subset *F' *⊆ *F *of factors and the set *F*(*S*), then *F' *is a *factorization agreement set *for *F*(*S*) if and only if for each sequence *s *∈ *S*, there exists a f-composition *f *in *F*(*s*) whose factor set is a subset of *F'*, i.e. *F*(*f*) ⊆ *F'*.

The *Minimum Factorization Agreement *problem, given a set *F *of factors and a set *S *of sequences, asks for a minimum cardinality subset *F' *⊆ *F *such that *F' *is a factorization agreement set for *F*(*S*). Then the CG problem can be reduced to the MFA problem by posing *S *to be the cluster of transcript sequences *P_i _*and *F *is the set of all genomic factors (exons) used to produce the compositions *C*(*P_i_*) for each *P_i_*, i.e. *F*(*S*) consists of all the compositions of each sequence in *S*. Then the consensus gene structure consists of a minimum factorization agreement set for the set of compositions of the transcripts data. When solving the MFA problem on such data, the solution *F' *provides a minimum set of factors explaining all transcript sequences and a single composition of each transcript can be obtained from set *F'*.

By applying the algorithm in [17] we can filter efficiently a set of spliced alignments agreeing to the same gene structure that are successively refined by the intron reduction step.

### Intron reduction

Although the intron boundaries of the EST spliced compositions are computed by finding the best transcript-genome alignment over the splice site regions and the most frequent intron pattern (i.e. the first and the last two nucleotides of an intron) according to [23], the set of predicted introns may still contain false positives very close to true predictions. Thus, we designed a procedure for comparing the intron set computed by the EST spliced compositions in order to correct and reduce the set of false positives.

In the following, let the pair (*i*, *s*) denotes a genomic intron (eventually specified by a pair of genomic coordinates) and a spliced composition of an EST *s *supporting the intron *i*, i.e. the composition has two consecutive factors *f_j_*, *f_j_*_+1 _inducing intron *i *when aligned to the genome. Then, given an error bound *b*, we say that (*i*, *s*) is *b*-reducible to (*i'*, *s*) iff there exists a boundary shift of factors *f_j _*and *f_j_*_+1 _of a new spliced composition of *s *inducing intron *i' *with at most *b *additional errors with respect to the previous alignment of the two factors against the genome. To improve the accuracy of the step, we also consider if the intron is supported by a RefSeq transcript and if it can be categorized as an U12/U2 intron. A RefSeq sequence is a validated full-length mRNA stored and annotated in the NCBI RefSeq database. U2 and U12 refers to two intron categories for which the excision is mediated by the major spliceosomal pathway or the minor spliceosomal pathway, respectively. Notice that RefSeq transcripts are usually full-length and error-free, that *GT *- *AG*, *GC *- *AG *and *AT *- *AC *are the most frequent rules [23] and those rules are associated to U12/U2 introns [24]. Hence we assume that only introns that do not follow one of the U12/U2 rules and are not supported by a RefSeq transcript should be reduced. The input of our intron-reduction procedure is a set *X *of pairs (*i*, *s*) computed by the previous steps. Then, *R *is the set of pairs in *X *such that *s *is a RefSeq, *C*_1_, *C*_2_, *C*_3 _and *N *are the set of pairs in *X *\ *R *following the *GT *- *AG*, *GC *- *AG*, *AT *- *AC *and a non-U12/U2 rule respectively. Our procedure basically tries to reduce elements in *N *to some intron in *R *and, if this is not possible, it tries to reduce to some element in the first set of the sequence *C*_1_, *C*_2_, *C*_3 _that allows the reduction.

## Results

We implemented the approach described in the previous section as a set of programs in the software package PIntron. PIntron receives a genomic sequence and a set of transcripts - ESTs and/or mRNAs - and computes a representation of the exon-intron structure of the gene as well as a set of predicted full-length annotated isoforms. PIntron outputs the list of the predicted introns with information such as relative and absolute start and end positions, intron lengths, the donor and the acceptor splice sites, and intron types (U12, U2 or unclassified). The output gives the composition as exons of each isoform and, for each exon, the start and end positions as relative and absolute coordinates, if a polyA signal is present, and the length of 5'UTR and 3'UTR. Moreover several additional information are given for each predicted isoform, such as its length, the CDS starting and ending positions, the RefSeqID (if it exists) and the length of the associated protein.

PIntron source code and binaries are available under the GNU AGPLv3 license at http://www.algolab.eu/PIntron.

In the following, we discuss an experimental *in-silico *analysis on real human data aiming to evaluate our approach. Such an experimental evaluation is organized in two parts. The first part has been designed to assess the prediction accuracy of PIntron, while the aim of the second part is to show the scalability of our method and its effectiveness on genes that are very large or complex and are currently outside the comfort zone of the most used methods.

We have assessed the accuracy achieved by PIntron by comparing it with ASPic [20] and Exogean [21]. In particular, ASPic is a well-established software to predict alternative isoforms by multiple EST/mRNA alignments against the corresponding genomic regions. For each input EST, the ASPic algorithm attempts to compute a single spliced alignment with the minimum number of exons. Instead, PIntron implicitly provides several candidate spliced alignments for each EST, among which the best one is selected by using the MFA agreement approach, thus allowing a greater accuracy in predicting the putative gene structure. Moreover, PIntron is much faster than ASPic because of the more efficient data structure used for performing the EST alignments (i.e. the embedding graph instead of the hash table of the genomic seeds employed by ASPic). For this reason, ASPic requires a genomic sequence trimmed at the borders of a single gene locus, while PIntron is able to efficiently process a large region of the genome (i.e. spanning tens of gene loci) and a large set of expressed sequences.

Exogean is a gene prediction tool based on pre-aligned (by Blat [25]) ESTs/mRNAs or proteins. Exogean resulted one of the most accurate gene finding system in the last EGASP competition [26]. In Exogean, gene structures are reconstructed according to a graph-based strategy mimicking the human annotation process.

The accuracy assessment has been performed on 13 ENCODE human regions [26] used as training set in the EGASP competition. The regions have been chosen since they present different gene density and different conservation to the mouse genome. This dataset contains 112 well-annotated gene loci, supported by 98, 064 UniGene transcripts for a overall length of approximately 62 Mb (Table 1). The 13 ENCODE regions represent, approximately, 8.5 Mb of the human genomic sequence. Supplementary Table S.1 in Additional file 1 reports the complete list of the genes used in this experimental evaluation along with some of their main characteristics. ESTs and mRNAs related to each gene were obtained from UniGene database.

**Table 1:** Main characteristics of the dataset used for the accuracy assessment of PIntron

Region	Genomic length (nt)	Number of genes	Number of transcripts	Overall transcript length (nt)
ENm004	1,700,000	18	6,964	4,497,709
ENm006	1,338,447	35	18,230	11,377,148
ENr111	500,000	2	171	113,356
ENr114	500,000	1	35	120,734
ENr132	500,000	4	855	551,266
ENr222	500,000	2	461	277,554
ENr223	500,000	5	50,607	32,732,634
ENr231	500,000	11	5,637	3,534,406
ENr232	500,000	9	4,779	2,505,934
ENr323	500,000	5	1,670	997,647
ENr324	500,000	1	487	343,220
ENr333	500,000	12	7,179	4,381,534
ENr334	500,000	7	989	611,795

Total	8,538,447	112	98,064	62,044,937

The results of our first assessment are summarized in Table 2, while the details are presented in Supplementary Tables S.2, S.3, S.4 in Additional file 1. The three tools have been evaluated according to two dimensions: prediction quality and time efficiency. The first important observation is that only PIntron was able to predict the gene structures for all 112 ENCODE loci, while ASPic and Exogean completed 93 and 104 genes, respectively. Moreover, PIntron has been the fastest of the three in the experiment over the whole set of genes, producing its results in about 49 minutes (on average 26 seconds per gene). On the genes that have been successfully processed, instead, Exogean took 57 minutes and ASPic more than 46 hours. Such results clearly indicate a computational improvement of PIntron over Exogean and especially ASPic in processing genes that are critical in terms of number of ESTs. Indeed Table 3 shows that PIntron scales much better than Exogean and ASPic when the number of transcripts is over 10,000, thus making our new software implementation particularly amenable to analyze large EST clusters. Notice that the running time of Exogean includes the preprocessing time required by Blat to align the transcripts. However, the preprocessing time is almost negligible compared to the time required by Exogean. In fact, Blat required approximately 4 minutes (7% of the total running time) to process all the genes.

**Table 2:** Summary of the experimental results on the **112 **gene loci on the **13 **ENCODE regions

		PIntron	Exogean	ASPic
Exon level	Sn	**0.529**	0.444	0.390
	Sp	**0.622**	0.606	0.427

Intron level	Sn	**0.874**	0.733	0.633
	Sp	**0.789**	0.777	0.567

Transcript level	Sn	**0.564**	0.251	0.342
	Sp	0.418	**0.450**	0.252

Nucleotide level	Sn	**0.889**	0.657	0.635
	Sp	**0.916**	0.865	0.632

Annotated genes		**112**	104	93

Total running time (seconds)		**2,961**	3,446	168,607

The best value of each row is highlighted in boldface.

**Table 3:** Running times of PIntron and Exogean on the 26 "critical" genes

Gene	Genomic length (nt)	Number of transcripts	Running time (seconds)	
			
			PIntron	Exogean
ACTB	36,634	26,248	287.35	371.22
ALB	24,299	16,920	144.17	369.38
ANKS1B	1,258,645	406	15.60	0.92
ANXA1	512,535	2,087	20.65	7.63
ATP1A1	619,226	3,241	27.82	11.90
ATP5A1	405,213	9,864	143.33	70.93
CDH13	1,169,823	507	10.34	1.02
CNTNAP2	2,304,964	227	30.86	1.01
CTNNA2	1,463,710	261	12.71	0.96
CUGBP2	1,081,163	864	18.04	2.42
DAB1	1,551,956	164	14.51	0.85
DLG2	2,172,263	279	21.18	1.15
DMD	2,241,933	329	35.35	2.21
ENO1	185,661	13,131	119.84	125.51
FGG	579,042	2,033	15.40	3.56
FHIT (^†^)	1,502,110	134	202.35	n.a.
GAPDH	46,975	15,518	149.64	232.81
HINT1	873,331	844	12.02	3.08
HSP90AA1	384,611	6,710	47.37	13.87
HSPA8	90,642	15,850	118.47	152.84
KCNIP4	1,220,613	107	10.09	0.65
MBP	154,857	21,071	251.70	1,344.42
NCAM1	317,404	1,293	12.54	1.63
RPL3	187,677	12,208	90.15	108.12
TBC1D22A	1,378,585	467	115.99	2.27
TTN	304,814	1,349	1,952.58	6.77

Total	22,068,686	152,112	3,880.05	2,837.94

^† ^Exogean did not successfully compute a gene structure for FHIT.

**Figure 4:**
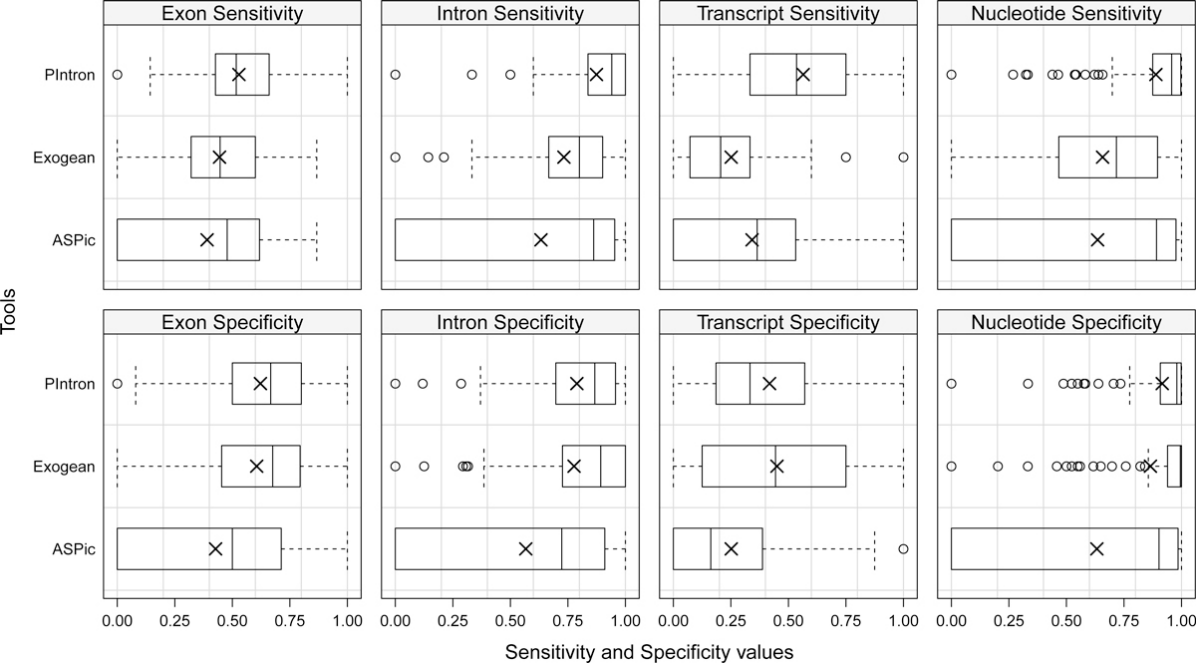
**Accuracy achieved by PIntron, Exogean and ASPic at various levels**. The boxplot presents the distribution of specificity and sensitivity achieved by the three tools at the exon, intron, transcript and nucleotide levels. The vertical edges of the boxes represent the first quartile, the median and the third quartiles (from left to right). The cross is the average. The vertical dashed lines represent an estimate of the 95% confidence interval of the median. The circles are all the outliers with respect to such confidence interval.

Prediction quality has been evaluated by calculating sensitivity (Sn) and specificity (Sp) between ENCODE annotations and predictions at nucleotide, exon, intron, and transcript level, according to Burset and Guigó [27]. We adhered to the nomenclature established in the literature aimed to the evaluation of gene structure prediction tools, even if the definition of specificity that we use here is called positive predictive value in statistical literature [28]. As shown in Table 2 and Figure 4, PIntron appears the most accurate program at diverse prediction levels. Moreover, PIntron exhibits sensitivity and specificity levels that are quite similar. This fact, which is highly desirable in any prediction tool, shows that PIntron does not advantage any of them to the detriment of the other one. In addition, our results (see the average sensitivity at transcript level in Table 2) indicate that PIntron improves the reconstruction of exact transcripts when compared with ASPic and Exogean. Moreover, we want to recall that PIntron has completed the analysis of all 112 input genes, while Exogean and ASPic did not complete the task for 8 and 29 genes respectively.

Our second experimental analysis is devoted to evaluating the efficiency and the scalability of our approach on a subset of *critical *human genes that are particularly hard to analyze with the currently available programs because those genes have (1) a particularly complex gene structure (several tens of exons), or (2) a particularly large cluster of expressed sequences, or (3) a large genomic sequence.

To this aim, we selected 26 "critical" genes and we processed them with PIntron and Exogean on a 4-node linux cluster running CentOS 5.5. Each node is equipped with a quad-core 2.40 GHz CPU and 32 GiB of RAM. The genomic sequence has an average length of about 848 Kb, and is longer than 1 Mb for 11 of the 26 genes. Moreover, the selected genes have on average more than 5,000 transcripts, and 5 genes have more than 15,000 transcripts. The total running time was 65 minutes for PIntron and 48 minutes for Exogean. In this evaluation, we did not take into account ASPic since it was not able to give a solution for any of these genes within an acceptable time. Table 3 reports the complete list of genes considered in this experimental part along with their main characteristics and the running times of PIntron and Exogean. While Exogean and PIntron running times were both acceptable, PIntron averaged 149 sec/gene and Exogean 109 sec/gene. This is remarkable, since Exogean is based on the fast progressive EST-to-genome mapping program Blat and does not take into account potential alignment errors at splicing sites which, in turn, is likely to result in predictions that are not as accurate as those given by PIntron. The comparison of running times confirms our previous observation: PIntron, although slower than Exogean on genes with small transcript clusters, scales significantly better than Exogean when the cluster size increases. In fact, PIntron was systematically faster than Exogean on the subset of genes whose transcript cluster is composed by more than 10, 000 sequences (genes *ACTB*, *ALB*, *ENO1*, *GAPDH*, *HSPA8*, *MBP*, and *RPL3*), while it was slower than Exogean on the other genes. In almost all the cases where PIntron was slower than Exogean, the difference between the running times of the two tools is small. Thus the running time of PIntron can be considered acceptable also on these genes. One notable exception is gene *TTN *where PIntron took about 32 minutes to predict the gene structure, while Exogean required only a few seconds. The likely reason is that the input transcript set of *TTN *contains sequences that are more than 80 Kb long. Since EST sequences have a lower quality than mRNA sequences, computing their spliced alignment requires a considerable amount of computational resources.

We want to point out that our second experiment has limited scope. In fact a complete comparison of PIntron and Exogean would also include the accuracy dimensions. The results of the first experiment suggests that PIntron is more accurate than Exogean. If confirmed, the greater accuracy would justify the small increase in the running times that we have observed.

The analysis of the running times of the first and the second part of the experimentation has not shown any significant correlation between the length of the genes and the running times, hence confirming our conjecture that the behavior of our algorithm depends on some properties of the Embedding Graph, and not on the size of the instance. In particular, the structure of the Embedding Graph is strictly related to the quality of the transcripts and to the presence of repetitions and highly duplicated regions in the genomic sequence that, in turn, could influence the size of the graph. Also these results have confirmed our beliefs, since the average running time of the second experiment (149 sec/gene) is not too far from the running times on the smaller genes of the first experiment, where the average value is 26 sec/gene. A fundamental observation is that PIntron has successfully completed the analysis of all 26 "critical" genes, while Exogean did not complete the analysis for *FHIT*.

## Conclusions

In this work, we presented a new computational pipeline - PIntron - for predicting the gene structure into exons and introns from a cluster of transcript (EST, mRNA) sequences. PIntron combines two ideas: a novel algorithm of proved small time complexity for computing spliced alignments of a transcript against a genome, and an efficient algorithm that exploits the inherent redundancy of information in a cluster of transcripts to select, among all possible factorizations of EST sequences, those allowing to infer splice site junctions that are largely confirmed by the input data. PIntron is freely available at http://www.algolab.eu/PIntron under GNU Affero General Public Licence (AGPL). The experimental evaluation of PIntron has shown that it has been able to compute accurate predictions (whose level is comparable with that of other prediction tools) while achieving a good scalability to critical genes, especially if associated with a large transcript cluster.

## Competing interests

The authors declare that they have no competing interests.

## Authors' contributions

YP and RR designed the algorithm, developed the pipeline, designed and helped to perform the experiments, and drafted the manuscript. EP helped to design and to perform the experiments, and interpreted the results. GP helped to design the experiments and supervised the interpretation of the results. GDV helped to design the algorithm, to develop the pipeline, and to draft the manuscript. PB designed the algorithm, helped to draft the manuscript, and supervised the research. All authors read and approved the final manuscript.

## Supplementary Material

Additional file 1 **Supplementary tables**. Characteristics of the first dataset and detailed results obtained in the experimental comparison. Click here for file
