# Supplementary material for: PIntron: a fast method for detecting the gene structure due to alternative splicing via maximal pairings of a pattern and a text
Source: BMC Bioinformatics. 2012 Apr 12;13(Suppl 5):S2. doi: 10.1186/1471-2105-13-S5-S2 (PMC3358663; doi:10.1186/1471-2105-13-S5-S2)
Supplement: Additional file 1 — Supplementary tables. Characteristics of the first dataset and detailed results obtained in the experimental comparison. [file 1471-2105-13-S5-S2-S1.pdf]

PIntron: a fast method for detecting the gene structure due to alternative splicing via maximal pairings of a pattern and a text

Supplementary Material

Yuri Pirola[1,2 †], Raffaella Rizzi[1 †], Ernesto Picardi[3], Graziano Pesole[3,4], Gianluca Della Vedova[5] and Paola Bonizzoni[1*]

[1]Dip. di Informatica Sistemistica e Comunicazione, Univ. degli Studi di Milano–Bicocca, Milan, 20126, Italy.
[2]Centro Ricerche e Studi Agroalimentari, Parco Tecnologico Padano, Lodi, 26900, Italy.
[3]Dip. di Biochimica e Biologia Molecolare "E. Quagliariello", Univ. degli Studi di Bari, Bari, 70126, Italy.
[4]Istituto di Biomembrane e Bioenergetica, Consiglio Nazionale delle Ricerche, Bari, 70126, Italy.
[5]Dip. di Statistica, Univ. degli Studi di Milano–Bicocca, Milan, 20126, Italy.

Email: Yuri Pirola - yuri.pirola@disco.unimib.it; Raffaella Rizzi - raffaella.rizzi@unimib.it; Ernesto Picardi - e.picardi@biologia.uniba.it; Graziano Pesole - graziano.pesole@biologia.uniba.it; Gianluca Della Vedova - gianluca.dellavedova@unimib.it; Paola Bonizzoni*- bonizzoni@disco.unimib.it;

*Corresponding author

†These authors have equally contributed to the work and should therefore be considered jointly first authors.

List of Tables

S.1 Characteristics of the first dataset. 2
S.2 Results obtained by PIntron on the first dataset. 5
S.3 Results obtained by Exogean on the first dataset. 8
S.4 Results obtained by ASPic on the first dataset. 11

Suppl. Table S.1: Characteristics of the first dataset, which is composed by 112 gene loci on 13 ENCODE regions.

Region	Gene symbol	Genomic length (nt)	No. of transcripts	Total transcript length (nt)
ENm004	BPIL2	1700000	17	13862
	C22orf24	1700000	16	10324
	C22orf28	1700000	1308	845251
	C22orf30	1700000	204	127010
	DEPDC5	1700000	159	141907
	DRG1	1700000	532	324422
	EIF4ENIF1	1700000	302	182565
	FBXO7	1700000	781	550081
	PISD	1700000	459	285225
	RFPL2	1700000	24	23204
	RFPL3	1700000	12	12785
	RFPL3S	1700000	55	30933
	SFI1	1700000	294	199864
	SLC5A1	1700000	89	59849
	SLC5A4	1700000	8	7059
	SYN3	1700000	69	55662
	TIMP3	1700000	1700	1030314
	YWHAH	1700000	935	597392
ENm006	ARHGAP4	1338447	270	159666
	ATP6AP1	1338447	1936	1158050
	AVPR2	1338447	24	27508
	BRCC3	1338447	162	102709
	CTAG1A	1338447	13	8814
	CTAG1B	1338447	13	8814
	CTAG2	1338447	35	24526
	DKC1	1338447	553	355262
	DNASE1L1	1338447	207	133868
	EMD	1338447	334	209989
	F8	1338447	152	143445
	F8A1	1338447	205	138750
	FAM3A	1338447	394	274684
	FAM50A	1338447	367	211241
	FLNA	1338447	2289	1421838
	FUNDC2	1338447	369	226531
	G6PD	1338447	618	406006
	GAB3	1338447	79	60140
	H2AFB1	1338447	6	2716
	HCFC1	1338447	339	208273
	IKBKG	1338447	295	214614
	IRAK1	1338447	679	477941
	L1CAM	1338447	332	250684
	LAGE3	1338447	149	62001
	MECP2	1338447	394	308188
	MPP1	1338447	542	334002

(continue)

Suppl. Table S.1: Characteristics of the first dataset.

Region	Gene symbol	Genomic length (nt)	No. of transcripts	Total transcript length (nt)
	MTCP1	1338447	160	84001
	OPN1LW	1338447	14	9241
	OPN1MW	1338447	10	10398
	PLXNA3	1338447	166	93421
	RENBP	1338447	56	38929
	RPL10	1338447	6414	3770575
	SLC10A3	1338447	127	99164
	TAZ	1338447	240	167507
	TKTL1	1338447	287	173652
ENr111	KATNAL1	500000	141	94045
	RP11-374F3.4	500000	30	19311
ENr114	PCDH15	500000	35	120734
ENr132	ATP11A	500000	396	247284
	F10	500000	93	67228
	F7	500000	34	28716
	MCF2L	500000	332	208038
ENr222	ENPP1	500000	242	144392
	MOXD1	500000	219	133162
ENr223	C6orf150	500000	46	37102
	DDX43	500000	46	35091
	EEF1A1	500000	49936	32280756
	KCNQ5	500000	89	97648
	MTO1	500000	490	282037
ENr231	CGN	500000	204	128306
	PIP5K1A	500000	479	290946
	POGZ	500000	384	266137
	PSMB4	500000	1428	923678
	PSMD4	500000	709	440877
	RFX5	500000	496	304083
	SELENBP1	500000	845	502546
	SNX27	500000	373	209476
	TUFT1	500000	164	116073
	VPS72	500000	396	242030
	ZNF687	500000	159	110254
ENr232	C9orf106	500000	9	8499
	CRAT	500000	324	203771
	DOLPP1	500000	197	122147
	FAM73B	500000	537	305027
	IER5L	500000	164	88796
	NUP188	500000	512	369440
	PHYHD1	500000	127	83097
	PPP2R4	500000	1387	833497
	SH3GLB2	500000	1522	491660

(continue)

Suppl. Table S.1: Characteristics of the first dataset.

Region	Gene symbol	Genomic length (nt)	No. of transcripts	Total transcript length (nt)
ENr323	LACE1	500000	46	33074
	NR2E1	500000	37	32825
	OSTM1	500000	307	189503
	SEC63	500000	537	297320
	SNX3	500000	743	444925
ENr324	STAG2	500000	487	343220
ENr333	C20orf173	500000	19	10781
	CEP250	500000	211	149928
	CPNE1	500000	1675	1065143
	ERGIC3	500000	1235	694708
	FAM83C	500000	16	16202
	FER1L4	500000	100	67001
	GDF5	500000	41	35466
	MMP24	500000	86	58264
	NFS1	500000	553	331736
	RBM12	500000	1675	1065143
	RBM39	500000	1524	864908
	SPAG4	500000	44	22254
ENr334	FOXP4	500000	182	141106
	FRS3	500000	60	42640
	MDFI	500000	115	75705
	NCR2	500000	25	14807
	PGC	500000	377	187560
	TFEB	500000	211	131431
	USP49	500000	19	18546

Suppl. Table S.2: Results obtained by PIntron on the first dataset. The time is in seconds.

Gene symbol	Sn_e	Sp_e	Sn_i	Sp_i	Sn_t	Sp_t	Sn_n	Sp_n	Time
ARHGAP4	0.479	0.739	0.767	0.917	0.333	0.163	0.896	0.86	14.07
ATP11A	0.694	0.81	0.971	0.892	0.5	0.357	0.856	0.999	18.04
ATP6AP1	0.571	0.4	1	0.6	0.8	0.308	0.541	0.942	23.89
AVPR2	0.444	0.5	1	1	0.667	0.5	0.998	0.999	8.91
BPIL2	0.895	0.81	0.941	0.727	0.5	0.2	0.982	0.961	9.87
BRCC3	0.6	0.833	0.938	0.882	0.714	0.385	1	0.893	11.62
C20orf173	0.455	0.294	1	0.462	0.333	0.167	0.933	0.487	2.98
C22orf24	0.75	0.857	1	1	1	0.75	1	1	8.26
C22orf28	0.481	0.464	0.867	0.684	0.375	0.231	0.974	0.985	18.49
C22orf30	0.5	0.917	0.923	0.923	0.714	0.714	1	1	34.87
C6orf150	0.455	0.556	0.833	1	0.5	0.667	0.582	0.982	8.91
C9orf106	0.5	0.5	1	1	1	1	0.268	1	21.39
CEP250	0.667	0.717	0.953	0.804	0.3	0.067	0.987	0.907	5.10
CGN	0.742	0.742	0.917	0.917	0.714	0.5	0.973	0.933	5.63
CPNE1	0.359	0.56	0.75	0.714	0.172	0.033	0.878	0.333	17.75
CRAT	0.485	0.696	0.826	0.864	0.222	0.095	0.848	0.98	5.12
CTAG1A	0.6	0.429	1	0.5	1	0.667	0.988	0.582	9.14
CTAG1B	0.8	1	1	1	1	1	0.988	1	26.41
CTAG2	0.8	0.333	1	0.4	0.5	0.2	1	0.735	10.46
DDX43	0.81	0.773	1	0.895	0.667	0.333	0.999	0.938	3.12
DEPDC5	0.667	0.771	0.963	0.897	0.211	0.071	0.904	0.912	27.09
DKC1	0.5	0.731	0.857	0.947	0.417	0.263	0.924	0.992	13.88
DNASE1L1	0.565	0.684	0.857	0.923	0.5	0.364	0.87	0.874	12.44
DOLPP1	0.571	0.667	0.9	0.818	0.75	0.429	0.998	0.959	4.70
DRG1	0.545	0.429	1	0.583	0.667	0.4	0.929	0.795	14.59
EEF1A1	0.171	0.163	0.5	0.286	0.133	0.048	0.439	0.987	726.48
EIF4ENIF1	0.6	0.706	0.964	0.964	0.273	0.034	0.993	0.893	16.62
EMD	0.346	0.643	0.875	0.875	0.8	0.727	0.961	1	16.11
ENPP1	0.826	0.826	0.947	1	0.333	0.5	0.906	1	195.02
ERGIC3	0.526	0.652	0.963	0.788	0.842	0.267	0.536	0.982	10.40
F10	0.143	0.333	1	1	0.5	1	1	0.549	2.46
F7	0.65	0.867	1	0.933	0.6	0.375	0.907	0.909	2.78
F8	0.771	0.871	1	0.966	1	0.833	0.995	0.97	124.70
F8A1	0	0	0	0	1	1	0.993	1	14.97
FAM3A	0.364	0.571	0.895	0.81	0.5	0.321	0.937	0.993	25.79
FAM50A	0.714	0.5	1	0.684	0.333	0.1	0.956	0.837	14.71
FAM73B	0.512	0.595	0.929	0.788	0.692	0.243	0.978	0.915	15.75
FAM83C	0.75	0.545	1	0.75	1	0.5	1	0.929	2.86
FBXO7	0.458	0.478	0.933	0.667	0.5	0.094	0.699	0.969	20.89
FER1L4	0.592	0.882	0.804	0.957	0.231	0.214	0.87	0.982	6.67
FLNA	0.584	0.693	0.867	0.881	0.105	0.03	0.988	0.97	25.98
FOXP4	0.759	0.846	0.952	0.87	0.4	0.2	0.999	1	58.70
FRS3	0.615	0.889	0.727	1	0.25	0.333	0.908	1	2.69
FUNDC2	0.474	0.692	1	0.909	0.714	0.625	0.639	1	13.77
G6PD	0.677	0.636	0.941	0.8	0.4	0.129	0.959	0.981	12.89
GAB3	0.588	0.714	0.929	1	0.75	0.429	0.93	0.983	20.92

(continue)

Suppl. Table S.2: Results obtained by PIntron on the first dataset.

Gene symbol	Sn_e	Sp_e	Sn_i	Sp_i	Sn_t	Sp_t	Sn_n	Sp_n	Time
GDF5	0.8	0.667	1	1	1	1	1	0.998	29.50
H2AFB1	0	0	0	0	1	1	0.879	1	8.72
HCFC1	0.735	0.893	0.964	1	0.25	0.333	0.951	1	11.86
IER5L	0	0	0	0	1	1	0.999	1	5.78
IKBKG	0.405	0.517	0.81	0.607	0.429	0.3	0.464	0.576	16.35
IRAK1	0.271	0.5	0.69	0.833	0.2	0.267	0.885	0.98	28.12
KATNAL1	0.632	0.8	1	1	0.4	0.5	0.966	1	83.64
KCNQ5	0.737	0.875	0.938	0.882	0.667	0.571	0.952	0.999	8.02
L1CAM	0.603	0.795	1	0.949	0.467	0.152	0.959	0.993	12.13
LACE1	0.5	0.778	0.636	1	0.2	0.5	0.782	0.978	5.18
LAGE3	1	0.6	1	0.667	1	0.5	1	0.523	13.32
MCF2L	0.469	0.662	0.857	0.889	0.5	0.281	0.622	0.928	6.76
MDFI	0.227	0.417	0.8	0.889	0.667	0.545	0.982	1	3.22
MECP2	0.333	0.45	0.684	0.722	0.5	0.538	0.87	0.858	29.28
MMP24	0.667	0.667	1	1	1	1	1	0.998	3.50
MOXD1	0.571	0.727	0.889	1	0.333	0.333	1	0.999	4.79
MPP1	0.4	0.714	0.957	0.957	0.611	0.268	0.981	0.994	13.54
MTCP1	0.471	0.667	1	0.917	1	0.875	1	0.997	12.32
MTO1	0.5	0.486	1	0.645	0.545	0.133	0.332	0.919	12.40
NCR2	0.636	0.875	1	1	1	1	1	1	2.24
NFS1	0.514	0.655	0.955	0.875	0.692	0.6	0.768	0.998	8.67
NR2E1	0.667	0.909	0.909	1	0.75	1	0.941	0.987	6.95
NUP188	0.8	0.839	1	0.939	0.545	0.13	0.99	0.998	36.61
OPN1LW	0.333	0.3	0.667	0.571	0	0	0.782	0.705	10.53
OPN1MW	0	0	0	0	0	0	0	0	11.71
OSTM1	0.429	0.75	0.643	1	0.571	1	0.895	0.995	45.81
PCDH15	0.75	0.811	0.738	0.838	0.176	0.25	0.98	0.994	5.01
PGC	0.625	0.526	0.909	0.714	0.25	0.143	1	0.775	4.66
PHYHD1	0.522	0.8	1	0.778	0.417	0.312	0.995	0.897	8.89
PIP5K1A	0.523	0.676	1	0.806	0.4	0.171	0.989	0.924	9.84
PISD	0.429	0.462	0.957	0.564	0.533	0.216	0.992	0.802	12.72
PLXNA3	0.787	0.902	0.97	1	0.5	0.333	0.998	0.986	10.14
POGZ	0.614	0.75	0.958	0.793	0.5	0.2	0.892	0.983	12.30
PPP2R4	0.385	0.476	0.964	0.711	0.625	0.205	0.997	0.929	43.31
PSMB4	0.545	0.667	1	0.778	0.714	0.5	1	1	13.64
PSMD4	0.368	0.452	0.778	0.583	0.231	0.073	0.886	0.978	10.68
RBM12	0.4	0.08	1	0.119	1	0.026	0.995	0.639	18.93
RBM39	0.355	0.465	0.923	0.766	0.625	0.12	0.873	0.837	20.31
RENBP	0.424	0.824	0.75	1	0.2	0.333	0.922	0.955	13.20
RFPL2	0.571	0.5	1	0.571	1	0.5	1	0.96	8.66
RFPL3	0.286	0.667	0.333	1	0.333	1	0.753	1	9.09
RFPL3S	0.5	0.5	1	0.556	0.75	0.5	0.871	0.845	8.76
RFX5	0.439	0.694	0.958	0.793	0.619	0.245	0.999	0.956	7.60
RP11-374F3.4	0.478	0.786	0.778	0.933	0.333	0.429	0.831	0.991	8.37
RPL10	0.303	0.233	1	0.37	0.733	0.216	0.998	0.79	86.54
SEC63	0.429	1	1	1	0.333	1	0.736	1	6.86
SELENBP1	0.408	0.541	0.955	0.724	0.353	0.167	0.838	0.989	8.90
SFI1	0.573	0.717	0.935	0.768	0.318	0.075	0.657	0.883	34.54

(continue)

Suppl. Table S.2: Results obtained by PIntron on the first dataset.

Gene symbol	Sn_e	Sp_e	Sn_i	Sp_i	Sn_t	Sp_t	Sn_n	Sp_n	Time
SH3GLB2	0.5	0.607	0.842	0.64	0.5	0.161	0.889	0.931	9.19
SLC10A3	0.364	0.444	0.6	0.6	0.75	0.5	0.959	0.993	10.19
SLC5A1	0.895	0.81	1	0.933	0.333	0.167	1	0.937	9.07
SLC5A4	0.867	0.867	1	1	1	1	0.995	0.999	8.50
SNX27	0.75	0.818	1	1	0.75	0.6	1	0.999	8.57
SNX3	0.5	0.5	1	0.636	1	0.571	0.997	0.944	7.18
SPAG4	0.52	0.591	0.933	0.933	0.429	0.3	0.956	0.962	3.22
STAG2	0.653	0.839	0.925	0.875	0.316	0.088	0.931	0.949	9.52
SYN3	0.419	0.464	0.905	0.826	0.4	0.308	0.764	0.986	8.95
TAZ	0.323	0.75	0.76	0.95	0.538	0.467	0.877	0.999	16.79
TFEB	0.636	0.808	0.952	0.952	0.562	0.22	0.893	0.922	23.26
TIMP3	0.444	0.2	0.833	0.5	0.667	0.286	0.999	0.813	37.12
TKTL1	0.667	0.64	0.938	0.75	0.571	0.333	0.939	0.996	10.90
TUFT1	0.609	0.56	0.933	0.609	0.429	0.15	0.939	0.872	71.86
USP49	0.286	0.667	0.833	1	0.6	1	0.324	0.999	219.28
VPS72	0.5	0.833	0.75	0.75	0.75	0.75	1	1	6.75
YWHAH	0.333	0.3	1	0.778	0.6	0.5	0.987	0.905	31.97
ZNF687	0.5	0.588	0.923	0.857	0.833	0.714	0.997	0.986	5.89

Suppl. Table S.3: Results obtained by Exogean on the first dataset. The running time is in seconds and includes the preprocessing time required by Blat to align the transcripts.

Gene symbol	Sn_e	Sp_e	Sn_i	Sp_i	Sn_t	Sp_t	Sn_n	Sp_n	Time
ARHGAP4	0.394	0.757	0.674	0.935	0.25	0.75	0.65	0.997	1.94
ATP11A	0.612	0.857	0.882	0.938	0.1	0.25	0.754	1	1.31
ATP6AP1	0.357	0.278	0.556	0.385	0	0	0.313	0.997	6.59
AVPR2	0.556	0.833	0.8	1	0.333	0.5	0.935	1	0.56
BPIL2	0.842	0.727	0.941	0.762	0	0	0.96	0.961	0.72
BRCC3	0.52	0.722	0.688	0.917	0	0	0.863	1	1.38
C20orf173	0.273	0.3	0.833	0.556	0	0	0.789	0.559	0.30
C22orf24	0.25	0.667	0.667	1	0.333	1	0.716	1	0.49
C22orf28	0.37	0.714	0.733	0.917	0.125	0.5	0.688	0.98	5.58
C22orf30	0.5	0.846	0.769	1	0.429	1	0.981	1	1.30
C6orf150	0.364	0.667	0.833	1	0.25	1	0.464	0.977	0.31
C9orf106	0.5	0.5	1	1	1	1	1	0.99	0.26
CEP250	0.667	0.809	0.884	0.905	0.2	0.286	0.946	0.987	1.63
CGN	0.613	0.864	0.833	0.952	0.143	0.5	0.77	0.993	1.02
CPNE1	0.333	0.591	0.725	0.906	0.138	0.235	0.629	0.698	6.40
CRAT	0.545	0.72	0.783	0.9	0.333	0.5	0.794	0.995	1.08
CTAG1A	0.4	0.333	1	0.4	0	0	0.748	0.5	0.56
CTAG1B	0.6	0.5	1	0.4	0	0	0.747	0.5	0.93
CTAG2	0.6	0.6	1	0.667	0.5	0.5	0.948	1	0.83
DDX43	0.714	0.714	0.941	0.889	0.333	0.333	0.894	0.931	0.38
DEPDC5	0.605	0.721	0.907	0.907	0.158	0.25	0.76	0.962	1.69
DKC1	0.395	0.789	0.762	0.941	0.083	0.333	0.63	0.998	4.28
DNASE1L1	0.391	0.6	0.786	0.846	0.125	0.25	0.634	1	1.64
DOLPP1	0.571	0.667	0.8	0.889	0.5	0.667	0.969	0.998	0.68
DRG1									
EEF1A1	0.073	0.107	0.375	0.316	0	0	0.224	1	3186.87
EIF4ENIF1	0.675	0.771	0.893	0.926	0.364	0.5	0.708	0.984	1.16
EMD	0.269	0.636	0.75	0.857	0.2	0.667	0.699	1	2.25
ENPP1	0.783	0.947	0.947	1	0.333	1	0.906	0.999	0.96
ERGIC3	0.298	0.773	0.667	1	0.211	1	0.262	0.993	4.05
F10	0.143	0.333	1	1	0.5	1	1	0.549	0.56
F7	0.45	0.818	0.643	0.9	0.4	0.667	0.824	0.999	0.64
F8	0.771	0.964	0.964	1	0.2	0.5	0.945	1	0.90
F8A1									
FAM3A	0.386	0.447	0.895	0.81	0.222	0.222	0.412	0.995	2.14
FAM50A	0.524	0.579	1	0.812	0	0	0.655	0.89	5.08
FAM73B	0.488	0.6	0.786	0.815	0.077	0.125	0.48	0.856	1.44
FAM83C	0.5	0.444	0.833	0.714	0	0	0.492	1	0.30
FBXO7	0.417	0.526	0.8	0.923	0.4	0.667	0.278	0.998	5.21
FER1L4	0.671	0.864	0.893	0.893	0.077	0.143	0.792	0.995	0.72
FLNA	0.528	0.758	0.817	0.891	0.053	0.125	0.818	0.997	15.48
FOXP4	0.759	0.815	0.952	0.909	0.6	0.5	1	1	0.91
FRS3	0.538	1	0.545	1	0.25	1	0.762	1	0.43
FUNDC2	0.316	0.857	0.5	1	0.286	1	0.321	1	1.42
G6PD	0.419	0.481	0.824	0.778	0.3	0.429	0.675	0.994	4.55

(continue)

Suppl. Table S.3: Results obtained by Exogean on the first dataset.

Gene symbol	Sn_e	Sp_e	Sn_i	Sp_i	Sn_t	Sp_t	Sn_n	Sp_n	Time
GAB3	0.647	0.733	0.929	1	0.75	0.75	0.93	0.998	0.76
GDF5	0.6	0.75	1	1	0.5	1	0.971	1	0.35
H2AFB1									
HCFC1	0.735	0.781	0.893	0.893	0	0	0.983	1	2.04
IER5L									
IKBKG	0.324	0.308	0.619	0.406	0.143	0.133	0.415	0.617	3.03
IRAK1	0.271	0.8	0.586	1	0.25	1	0.876	1	3.20
KATNAL1	0.632	0.857	0.923	1	0.6	1	0.956	0.991	0.66
KCNQ5	0.579	0.846	0.875	0.875	0.333	0.4	0.87	1	1.02
L1CAM	0.603	0.854	0.946	0.972	0.467	0.875	0.779	1	1.05
LACE1	0.357	0.714	0.455	0.833	0	0	0.676	0.975	0.37
LAGE3	0.667	0.4	1	0.667	1	0.5	1	0.523	1.26
MCF2L	0.375	0.8	0.679	0.974	0.125	0.444	0.28	0.977	1.21
MDFI	0.273	0.4	0.7	0.778	0.333	0.6	0.83	1	0.72
MECP2	0.074	0.333	0.211	1	0.143	1	0.69	1	1.52
MMP24	0.667	0.667	1	1	1	1	1	0.998	0.55
MOXD1	0.5	0.636	0.778	0.875	0.333	0.5	0.971	0.999	0.65
MPP1	0.5	0.758	0.826	0.905	0.222	0.444	0.443	1	2.16
MTCP1	0.412	0.778	0.636	1	0.143	0.5	0.496	1	0.69
MTO1	0.5	0.362	0.95	0.679	0.364	0.25	0.297	0.913	1.72
NCR2	0.818	0.9	1	1	1	1	0.888	1	0.75
NFS1	0.649	0.686	0.864	0.731	0.462	0.4	0.604	1	2.56
NR2E1	0.6	0.818	0.818	1	0.5	1	0.898	0.986	0.32
NUP188	0.723	0.758	0.978	0.9	0.273	0.375	0.711	0.999	3.47
OPN1LW	0.444	0.286	0.667	0.308	0	0	0.821	0.332	0.63
OPN1MW	0.444	0.222	0.833	0.294	0	0	1	0.202	0.67
OSTM1	0.333	0.778	0.5	1	0.286	1	0.869	0.995	0.70
PCDH15	0.675	0.844	0.714	0.968	0.176	0.6	0.535	0.989	0.42
PGC	0.562	0.75	0.727	0.8	0.25	0.5	0.867	0.758	0.97
PHYHD1	0.522	0.545	1	0.778	0.25	0.3	0.936	0.995	0.50
PIP5K1A	0.364	0.8	0.72	0.9	0.2	0.6	0.718	0.998	1.49
PISD	0.333	0.452	0.609	0.538	0.067	0.091	0.489	0.841	2.57
PLXNA3	0.681	0.97	0.97	1	0.125	1	0.903	1	2.25
POGZ	0.523	0.697	0.875	0.808	0.143	0.222	0.662	0.975	1.16
PPP2R4	0.365	0.528	0.714	0.833	0.25	0.462	0.663	0.926	2.63
PSMB4	0.318	0.333	0.857	0.545	0.143	0.167	0.447	1	4.61
PSMD4	0.263	0.455	0.611	0.786	0.231	0.5	0.47	0.997	2.93
RBM12	0.3	0.068	0.8	0.125	0	0	0.243	0.458	4.74
RBM39	0.269	0.625	0.769	0.882	0	0	0.359	1	4.79
RENBP	0.394	0.722	0.7	1	0.3	0.75	0.713	0.943	0.51
RFPL2	0.571	0.5	1	0.571	0.5	0.333	0.92	0.987	0.65
RFPL3	0.429	1	0.667	1	0.333	1	0.614	1	0.65
RFPL3S									
RFX5	0.316	0.643	0.667	0.842	0.143	0.3	0.877	0.993	1.44
RP11-374F3.4									
RPL10	0.152	0.263	0.6	0.429	0	0	0.171	0.649	69.94
SEC63									
SELENBP1	0.51	0.714	0.909	0.952	0.353	0.5	0.646	0.985	2.22

(continue)

Suppl. Table S.3: Results obtained by Exogean on the first dataset.

Gene symbol	Sn_e	Sp_e	Sn_i	Sp_i	Sn_t	Sp_t	Sn_n	Sp_n	Time
SFI1	0.467	0.603	0.761	0.778	0.045	0.083	0.36	0.936	1.55
SH3GLB2	0.471	0.516	0.895	0.81	0.2	0.222	0.274	0.99	4.72
SLC10A3	0.273	0.75	0.6	1	0.25	1	0.813	1	1.29
SLC5A1	0.737	0.824	1	0.933	0.333	0.5	0.984	0.97	0.79
SLC5A4	0.867	0.867	1	1	1	1	0.995	0.999	0.59
SNX27	0.333	1	0.333	1	0.25	1	0.301	1	0.43
SNX3	0.6	0.429	1	0.778	1	0.667	0.997	1	2.55
SPAG4	0.4	0.714	0.733	0.917	0.143	0.5	0.646	0.966	0.40
STAG2	0.569	0.788	0.83	0.957	0.158	0.375	0.875	0.998	1.43
SYN3	0.387	0.571	0.762	0.8	0	0	0.673	0.986	0.75
TAZ	0.215	0.667	0.64	0.842	0.077	0.222	0.322	1	2.35
TFEB	0.394	0.684	0.667	0.933	0.188	0.5	0.539	1	0.69
TIMP3	0.444	0.16	0.833	0.625	0.333	0.143	0.999	1	6.15
TKTL1	0.583	0.467	0.875	0.583	0.286	0.25	0.822	0.821	2.11
TUFT1	0.609	0.667	0.867	0.722	0.286	0.333	0.802	0.957	0.55
USP49	0.286	0.667	0.833	1	0.4	1	0.214	0.999	0.28
VPS72									
YWHAH	0.222	1	0.143	1	0.2	1	0.744	1	5.33
ZNF687	0.65	0.867	0.846	0.917	0.5	0.75	0.918	0.985	0.70

Suppl. Table S.4: Results obtained by ASPic on the first dataset. The time is in seconds.

Gene symbol	Sn_e	Sp_e	Sn_i	Sp_i	Sn_t	Sp_t	Sn_n	Sp_n	Time
ARHGAP4	0.479	0.773	0.744	0.914	0.333	0.148	0.879	0.901	76.00
ATP11A	0.776	0.809	0.971	0.892	0.5	0.333	0.859	0.992	1328.00
ATP6AP1	0.571	0.276	0.889	0.381	0	0	0.541	0.617	315.00
AVPR2	0.444	0.571	1	1	0.333	0.25	0.998	0.999	3.00
BPIL2	0.842	0.727	0.941	0.727	0.5	0.2	0.899	0.958	12.00
BRCC3	0	0	0	0	0	0	0.138	0.123	75.00
C20orf173									
C22orf24	0.75	0.857	1	1	1	0.75	1	1	3.00
C22orf28	0.519	0.519	0.867	0.765	0.625	0.294	0.984	0.913	420.00
C22orf30	0.545	0.8	0.923	0.923	0.714	0.714	1	1	3103.00
C6orf150	0.455	0.5	0.833	1	0.5	0.667	0.599	0.982	20.00
C9orf106	0.5	0.5	1	1	1	1	0.268	1	1.00
CEP250									
CGN	0.71	0.815	0.833	0.909	0.429	0.6	0.958	1	46.00
CPNE1									
CRAT	0.636	0.808	0.913	0.955	0.333	0.231	0.936	0.982	594.00
CTAG1A									
CTAG1B									
CTAG2									
DDX43	0.857	0.783	0.941	0.889	0.667	0.4	0.983	0.985	6.00
DEPDC5	0.667	0.74	0.944	0.895	0.158	0.068	0.909	0.91	367.00
DKC1	0	0	0	0	0	0	0	0	74.00
DNASE1L1	0.522	0.571	0.857	0.923	0.5	0.364	0.873	0.874	229.00
DOLPP1	0.5	0.636	0.9	0.9	0.75	0.75	0.967	0.998	16.00
DRG1	0.636	0.389	1	0.538	0.333	0.111	0.974	0.605	713.00
EEF1A1									
EIF4ENIF1	0.625	0.658	0.964	0.931	0.364	0.032	0.996	0.878	406.00
EMD	0.385	0.667	0.875	1	0.7	0.875	0.957	1	134.00
ENPP1	0.826	0.594	0.947	0.72	0	0	0.906	0.877	337.00
ERGIC3	0.526	0.732	0.926	0.893	0.684	0.181	0.542	0.984	167.00
F10	0.143	0.067	1	0.167	0.5	0.125	1	0.207	11.00
F7	0.65	0.812	0.929	0.929	0.6	0.375	0.901	0.909	5.00
F8	0	0	0	0	0	0	0.038	0.037	18010.00
F8A1									
FAM3A	0	0	0	0	0	0	0	0	61.00
FAM50A	0	0	0	0	0	0	0	0	42.00
FAM73B	0.605	0.591	1	0.737	0.615	0.211	0.998	0.901	142.00
FAM83C	0.5	0.444	0.5	0.6	0.5	0.333	0.978	0.928	3.00
FBXO7	0.458	0.44	0.933	0.7	0.5	0.111	0.647	0.961	99.00
FER1L4									
FLNA	0.562	0.685	0.867	0.912	0.211	0.058	0.998	0.965	1651.00
FOXP4	0.793	0.793	0.952	0.87	0.4	0.2	1	0.999	134.00
FRS3	0.615	0.667	0.909	0.909	0.5	0.5	0.978	1	9.00
FUNDC2	0	0	0	0	0	0	0	0	83.00
G6PD	0	0	0	0	0	0	0	0	138.00
GAB3	0	0	0	0	0	0	0	0	70.00

(continue)

Suppl. Table S.4: Results obtained by ASPic on the first dataset.

Gene symbol	Sn_e	Sp_e	Sn_i	Sp_i	Sn_t	Sp_t	Sn_n	Sp_n	Time
GDF5	0	0	0.333	1	0.5	1	0.899	0.998	10.00
H2AFB1									
HCFC1	0.735	0.781	0.964	1	0.25	0.25	0.951	0.96	104.00
IER5L									
IKBKG	0	0	0	0	0	0	0	0	370.00
IRAK1	0.322	0.514	0.655	0.76	0.2	0.148	0.904	0.972	154.00
KATNAL1	0.632	0.857	1	1	0.4	0.5	0.968	1	17021.00
KCNQ5	0.632	0.667	0.812	0.812	0.167	0.2	0.534	0.861	8988.00
L1CAM	0.621	0.735	0.973	0.947	0.4	0.13	0.945	0.993	298.00
LACE1	0.5	0.389	0.545	0.375	0	0	0.72	0.435	499.00
LAGE3	0	0	0	0	0	0	0	0	4.00
MCF2L	0.479	0.648	0.875	0.925	0.469	0.312	0.636	0.949	543.00
MDFI	0.364	0.571	0.8	0.889	0.667	0.545	0.993	1	23.00
MECP2	0.37	0.5	0.632	0.8	0.5	0.7	0.821	0.845	790.00
MMP24									
MOXD1	0.643	0.391	0.889	0.533	0	0	1	0.626	497.00
MPP1	0	0	0	0	0	0	0	0	245.00
MTCP1									
MTO1	0.5	0.5	1	0.667	0.455	0.114	0.332	0.916	116.00
NCR2	0.636	0.778	1	1	1	1	0.888	1	4.00
NFS1	0.541	0.69	0.909	0.833	0.538	0.5	0.771	0.998	183.00
NR2E1	0.8	1	1	1	0.75	0.75	1	1	5.00
NUP188	0.754	0.79	0.978	0.938	0.364	0.082	0.978	0.992	239.00
OPN1LW	0.556	0.833	0.833	1	0.5	1	1	0.983	1.00
OPN1MW	0.444	0.444	0.833	0.714	0.5	0.333	0.968	0.716	4.00
OSTM1	0.429	0.6	0.643	1	0.571	1	0.895	0.995	126.00
PCDH15									
PGC	0.5	0.444	0.818	0.692	0.25	0.167	0.867	0.733	50.00
PHYHD1	0.609	0.5	0.929	0.542	0.167	0.042	0.958	0.539	20.00
PIP5K1A	0.5	0.667	0.84	0.778	0.333	0.139	0.96	0.923	344.00
PISD	0.476	0.455	0.87	0.541	0.467	0.2	0.954	0.787	2167.00
PLXNA3	0	0	0	0	0	0	0	0	20.00
POGZ	0.659	0.763	0.917	0.786	0.429	0.214	0.882	0.985	229.00
PPP2R4	0.404	0.467	1	0.718	0.625	0.19	1	0.918	278.00
PSMB4	0.545	0.706	1	0.778	0.714	0.5	1	0.998	4684.00
PSMD4	0.421	0.457	0.833	0.652	0.385	0.132	0.888	0.966	86.00
RBM12									
RBM39	0.376	0.538	0.872	0.872	0.525	0.104	0.918	0.869	17546.00
RENBP	0.485	0.842	0.75	1	0.2	0.286	0.951	0.956	6.00
RFPL2									
RFPL3									
RFPL3S									
RFX5	0.474	0.711	1	0.828	0.619	0.2	0.999	0.956	40.00
RP11-374F3.4									
RPL10	0.303	0.25	1	0.417	0.733	0.22	0.872	0.762	55042.00
SEC63	0.571	0.105	1	0.067	0.333	0.029	1	0.095	1050.00
SELENBP1	0.408	0.556	0.955	0.778	0.471	0.229	0.845	0.986	140.00
SFI1	0.6	0.714	0.957	0.8	0.364	0.108	0.664	0.92	768.00

(continue)

Suppl. Table S.4: Results obtained by ASPic on the first dataset.

Gene symbol	Sn_e	Sp_e	Sn_i	Sp_i	Sn_t	Sp_t	Sn_n	Sp_n	Time
SH3GLB2	0.5	0.567	0.947	0.667	0.6	0.158	0.888	0.934	365.00
SLC10A3	0	0	0	0	0	0	0	0	11.00
SLC5A1	0.842	0.8	1	0.933	0.333	0.2	0.996	0.937	31.00
SLC5A4	0.867	0.867	1	1	1	1	0.995	0.998	3.00
SNX27	0.833	0.5	1	0.6	0	0	1	0.209	355.00
SNX3	0.5	0.455	1	0.778	1	0.667	0.997	1	1889.00
SPAG4	0.6	0.652	1	1	0.714	0.5	0.956	0.982	2.00
STAG2	0.639	0.719	0.83	0.83	0.368	0.171	0.948	0.976	14628.00
SYN3	0.452	0.609	0.81	0.85	0.2	0.25	0.703	0.987	4958.00
TAZ	0.338	0.786	0.76	0.95	0.538	0.583	0.971	1	42.00
TFEB	0.636	0.808	0.952	1	0.5	0.421	0.926	0.925	169.00
TIMP3	0.444	0.25	0.833	0.714	0.667	0.5	0.999	0.84	4046.00
TKTL1	0.625	0.75	0.875	0.824	0.429	0.375	0.932	1	29.00
TUFT1	0.652	0.517	0.867	0.619	0.571	0.267	0.994	0.878	93.00
USP49	0.429	0.545	0.833	0.556	0.4	0.5	0.309	0.86	42.00
VPS72	0.1	0.062	0.5	0.2	0.25	0.1	0.927	0.286	52.00
YWHAH	0.333	0.214	1	0.7	0.8	0.571	0.974	0.904	272.00
ZNF687	0.55	0.647	0.923	0.857	0.833	0.714	0.989	0.989	23.00
